# Medical Items

**Published:** 1912-05

**Authors:** 


					﻿‘itsJ .fagalloO	laarboK
Wto K ^)[W9^sT9Trft|i^aiice to
tft?' wen^ei’tf^ of ftKillciritf:fs irH^y'prox^^iyVratrfre^'^laborate
WiiVer, the
ftthVels^hfe k7cfrfeysl^nfl!'ttH8‘>^b'fft 'is'-file u<*ideal dlimifiktiVe” and
\(H11 ’1 iQoi^-fAfythbrbtf^ftly'1 e?xpeP Wv^dl^offS** W® Wave ac-
Bfftftti 1 at^fl rfh ‘f0ftf;r^^tefACTi^?ftl^ WbWflV* of sluggish feretory
- daoY ,B9vno2 is aabqalrq# aol vnoIoO
-fiijguHi £££ rffiW .yjaionft iargoloarnZ jIto'Z //sZ ydj lo
in •^y'pt&ctfe^hP Wbvya^Ptit fQity-"Sfid .particularly
during the summet^WtetP f'-ai^ulbtaftd /^t- fRichfidldu’Springs,
rgsbrtdffiWe^hottfandsbof r.tWsqmdtid and gouty ipaftients
tilteZf^^ulphWdE^ths^rfclwe1 prescribed ®engalin& e-xtfeansivel.y
AWd it'WaiWnlwdysnprdved most satisfactory/ n bna anoitsloqiat
’32B^s>Jf)vJ§tddfrstate abhatoowih^ tortheiearE -duck skiH >used, iurrats
l^sniifacvu^eq asbafeo.'dieoausefatJnsnalivc^yBrruniforffufandrr'igdweH
bemad bys'ttie:<Hllcfe>a«hpaT©ngaHife stands'! AonemostuanttQHgf a the
ready-made prescriptions for Rheumatism, neuralgia, grippe,
gout, etc. Besides the conscientious practitioner hesitates about
				

